# A comprehensive overview of *SMN* and *NAIP* copy numbers in Iranian SMA patients

**DOI:** 10.1038/s41598-023-30449-7

**Published:** 2023-02-24

**Authors:** Shahram Savad, Mahmoud Reza Ashrafi, Niusha Samadaian, Morteza Heidari, Mohammad-Hossein Modarressi, Gholamreza Zamani, Saloomeh Amidi, Sarang Younesi, Mohammad Mahdi Taheri Amin, Pourandokht Saadati, Alireza Ronagh, Hossein Shojaaldini Ardakani, Solat Eslami, Soudeh Ghafouri-Fard

**Affiliations:** 1 Genome-Nilou Laboratory, Tehran, Iran; 2 Pediatric Neurology Division, Children’s Medical Center, Pediatrics Center of Excellence, Ataxia Clinic, Tehran University of Medical Sciences, Tehran, Iran; 3 Department of Pediatrics Center, Growth and Development Research Center, Pediatrics Center of Excellence, Tehran University of Medical Sciences, Tehran, Iran; 4 Pediatric Neurology Division, Children’s Medical Center, Pediatrics Center of Excellence, Myelin Disorders Clinic, Tehran University of Medical Sciences, Tehran, Iran; 5 Department of Medical Genetics, School of Medicine, Tehran University of Medical Sciences, Tehran, Iran; 6 Prenatal Screening Department, Nilou Laboratory, Tehran, Iran; 7 Department of Pediatrics Neurologists, Shahid Bahonar Hospital, Alborz University of Medical Sciences, Karaj, Iran; 8 Department of Medical, Faculty of Medical Sciences, Alborz University of Medical Sciences, Karaj, Iran; 9 Department of Medical Biotechnology, School of Medicine, Alborz University of Medical Sciences, Karaj, Iran; 10 Dietary Supplements and Probiotic Research Center, Alborz University of Medical Sciences, Karaj, Iran; 11 Department of Medical Genetics, Shahid Beheshti University of Medical Sciences, Tehran, Iran

**Keywords:** Cell biology, Genetics, Molecular biology

## Abstract

Spinal muscular atrophy (SMA) is among the most common autosomal recessive disorders with different incidence rates in different ethnic groups. In the current study, we have determined *SMN1*, *SMN2* and *NAIP* copy numbers in an Iranian population using MLPA assay. Cases were recruited from Genome-Nilou Laboratory, Tehran, Iran and Pars-Genome Laboratory, Karaj, Iran during 2012–2022. All enrolled cases had a homozygous deletion of exon 7 of *SMN1*. Moreover, except for 11 cases, all other cases had a homozygous deletion of exon 8 of *SMN1*. Out of 186 patients, 177 (95.16%) patients showed the same copy numbers of exons 7 and 8 of *SMN2* gene. In addition, 53 patients (28.49%) showed 2 copies, 71 (38.17%) showed 3 copies and 53 patients (28.49%) showed 4 copies of *SMN2* gene exons 7 and 8. The remaining 9 patients showed different copy numbers of exons 7 and 8 of *SMN2* gene. The proportions of SMA patients with different numbers of normal *NAIP* were 0 copy in 73 patients (39.24%), 1 copy in 59 patients (31.72%), 2 copies in 53 patients (28.49%) and 4 copies in one patient (0.5%). These values are different from values reported in other populations. Integration of the data of the *SMN1*/*2* and *NAIP* genes showed 17 genotypes. Patients with genotype 0-0-3-3-1 (0 copies of *SMN1* (E7,8), 3 copies of *SMN2* (E7,8) and 1 copy of *NAIP* (E5)) were the most common genotype in this study. Patients with 0-0-2-2-0 genotype were more likely to have type I SMA. The results of the current study have practical significance, particularly in the genetic counseling of at-risk families.

## Introduction

Spinal muscular atrophy (SMA) is among the most common autosomal recessive disorders with an incidence rate of about 1 in 6000–10,000 live births. This disorder is described by degeneration of alpha motor neurons in the spinal cord and the medulla oblongata, leading to symmetrical proximal muscular atrophy. Heterozygous healthy carriers for this disorder have a frequency of 1 in 35 in the general population^1^. Based on the age of onset and reached motor functions, this disorder is classified into four clinical types, namely severe, intermediate, mild and adult-onset types being enumerated as types I to IV, respectively^2^. From a genetics point of view, this autosomal recessive disorder is caused by the dysfunction of the survival motor neuron (*SMN*) gene which is located on chromosome 5q13.2. This gene has two versions, namely *SMN1* and *SMN2*. The former produces a full-length transcript. These two versions are different from each other in only five nucleotides. Homozygous deletion of *SMN1* exon 7 is responsible for clinical disorder in approximately 94% of cases^3^. SMN2 has a partial function and can compensate homozygous deletions of *SMN1* to some extent^4^.Therefore, copy numbers of *SMN2* affect severity of SMA. Copy number of another gene located on chromosome 5q13.2, namely the neuronal apoptosis inhibitory protein (*NAIP*) gene has also been shown to be associated with severity of SMA^5^.

Variations in copy numbers of *SMN1* and *SMN2* have been reported in SMA patients from different populations. Moreover, different deletions and rearrangements have been detected in different ethnic groups^6–8^. Thus, identification of *SMN1*, *SMN2* and *NAIP* copy numbers in SMA patients in each population has a practical significance, particularly in the genetic counseling of at risk families. In the current study, we have determined *SMN1*, *SMN2* and *NAIP* copy numbers in an Iranian population of SMA patients using MLPA assay.

## Methods and patients

### Patients

A total of 186 SMA cases were enrolled in this study. Patients were referred to Genome-Nilou Laboratory, Tehran, Iran and Pars-Genome Laboratory, Karaj, Iran during 2012–2022. They were referred to Genome-Nilou laboratory by Iranian SMA Association and neurologists. They came from Tehran and other cities of Iran. All of them were genetically analyzed in Genome-Nilou laboratory. None of the patients used disease modifying therapies. All enrolled cases had a homozygous deletion of exon 7 of *SMN1* gene, as confirmed by MLPA assay. Ethical approval for this study has been obtained from the Ethical Committee of Tehran University of Medical Sciences. All methods were carried out in accordance with relevant guidelines and regulations. Informed consent forms were signed by all patients or their parents.

### MLPA assay

MLPA was performed using the SALSA MLPA Probemix P021-B1 for detection of deletions or duplication in the exons 7 and 8 of the SMN1, SMN2 and exon 5 of the NAIP genes (MRC-Holland, Amsterdam, Netherlands) as per the manufacturer’s instructions. The resulting fragments were separated using ABI PRISM 3100 (ThermoFisher Scientific, USA) and analyzed by GeneMarker software version 1.95^9^. Peak heights were normalized to control healthy individuals in a similar method to a previous study^10^, and a deletion or duplication was expected when the normalized peak ratio value was 0 (homozygous deletion), 1 (heterozygous deletion), 3 (heterozygous duplication) and occasionally 4 (heterozygous triplication or homozygous duplication). Each experiment included 4 controls; 2 normal controls, 1 carrier and 1 affected person. All of the controls had been confirmed in an external genetic laboratory.

### Statistical analyses

GraphPad Prism version 9.0 (GraphPad Software, La Jolla, CA, USA) (https://www.graphpad.com/guides/prism/latest/statistics/stat_checklist_kw.htm) was used for statistical analysis. Kruskal–Wallis test was performed to detect the relationship between copy number of exons 7 and 8 of SMN2 and deletion in exon 5 of NAIP gene, and SMA subtypes and age at onset. The quantitative data was expressed as mean ± standard deviation. The count data was expressed as the rate and frequency. P value less than 0.05 was considered statistically significant. To compare the distribution of clinical phenotypes (SMA subtypes) between patient groups with/without parental relationship, we used Chi-square (2 × 3 contingency table) (https://www.graphpad.com/quickcalcs/chisquared1.Chi-square/).

## Results

### General information

Based on the age of disease onset and clinical manifestations, 35, 47, 94 and 10 cases were classified as SMA types I–IV, respectively. A total of 114 cases (61.29%) were born to non-consanguineous parents. Others were born to first cousin (55 cases), first cousin once removed (14 cases) and second cousin (3 cases) parents. The study cohort included 83 females and 103 males.

### Gene copy numbers in SMA patients

All enrolled cases had a homozygous deletion of exon 7 of *SMN1*. Moreover, except for 11 cases, all other cases had a homozygous deletion of exon 8 of *SMN1*. Out of 186 patients, 177 (95.16%) patients showed the same copy numbers of exons 7 and 8 of *SMN2* gene. In addition, 53 patients (28.49%) showed 2 copies, 71 (38.17%) showed 3 copies and 53 patients (28.49%) showed 4 copies of *SMN2* gene exons 7 and 8. The remaining 9 patients showed different copy numbers of exons 7 and 8 of *SMN2* gene. The proportions of SMA patients with different numbers of normal *NAIP* were 0 copy in 73 patients (39.24%), 1 copy in 59 patients (31.72%), 2 copies in 53 patients (28.49%) and 4 copies in one patient (0.5%). Table 1 shows detailed characteristics of patients cohort.

**Table 1:** Detailed characteristics of patients cohort.

Case	Type	Sex	Age of onset	Age of diagnosis	Genes copy number					Parental relationship
					Exon7 SMN1	Exon8 SMN1	Exon7 SMN2	Exon8 SMN2	Exon5 NAIP	
1	I	Female	0M	3M	0	0	2	2	2	First cousins
2	I	Female	1M	3M	0	0	2	2	0	Not related
3	I	Female	2M	12M	0	0	2	2	0	First cousins
4	I	Female	2M	12M	0	0	2	2	0	First cousin once removed
5	I	Female	3M	6M	0	0	2	2	0	Not related
6	I	Female	3M	12M	0	0	2	2	0	First cousins
7	I	Female	3M	18M	0	0	2	2	0	First cousins
8	I	Female	4M	4M	0	0	2	2	0	First cousins
9	I	Female	4M	16M	0	0	2	2	0	First cousins
10	I	Female	4M	2Y	0	0	2	2	0	Not related
11	I	Female	5M	9M	0	0	2	2	0	First cousins
12	I	Female	5M	12M	0	0	2	2	0	Not related
13	I	Female	6M	6M	0	0	2	2	0	First cousins
14	I	Female	6M	8M	0	0	2	2	0	Not related
15	I	Male	0M	1M	0	0	2	2	0	First cousins
16	I	Male	1M	1M	0	0	2	2	0	First cousin once removed
17	I	Male	1M	2M	0	0	2	2	0	Not related
18	I	Male	1M	3M	0	0	2	2	2	First cousins
19	I	Male	1M	4M	0	0	2	2	0	Not related
20	I	Male	1M	6M	0	0	2	2	0	Not related
21	I	Male	2M	2M	0	0	2	2	1	First cousins
22	I	Male	2M	2M	0	0	2	2	0	Not related
23	I	Male	2M	4M	0	0	2	2	0	First cousin once removed
24	I	Male	2M	9M	0	0	2	2	0	First cousin once removed
25	I	Male	3M	4M	0	0	2	2	0	First cousin once removed
26	I	Male	3M	10M	0	0	2	2	0	Not related
27	I	Male	3M	10M	0	0	2	2	0	Not related
28	I	Male	3M	3Y	0	0	2	2	1	First cousins
29	I	Male	4M	4M	0	0	2	2	0	First cousins
30	I	Male	4M	6M	0	0	3	3	0	First cousins
31	I	Male	4M	9M	0	0	2	2	0	Not related
32	I	Male	5M	12M	0	0	2	2	0	First cousins
33	I	Male	6M	8M	0	0	3	3	2	First cousins
34	I	Male	6M	8M	0	0	4	4	0	First cousins
35	I	Male	6M	12M	0	0	2	2	1	First cousins
36	II	Female	6M	7Y	0	0	3	3	1	Not related
37	II	Female	6M	12Y	0	0	2	2	0	Not related
38	II	Female	9M	6Y	0	1	3	2	1	Not related
39	II	Female	10M	4Y	0	0	2	2	0	Not related
40	II	Female	11M	2Y	0	0	3	3	1	Not related
41	II	Female	11M	7Y	0	0	3	3	1	Not related
42	II	Female	11M	12Y	0	0	3	3	1	Not related
43	II	Female	12M	4Y	0	0	2	2	0	First cousins
44	II	Female	12M	5Y	0	0	2	2	0	First cousins
45	II	Female	12M	5Y	0	1	3	2	1	Not related
46	II	Female	12M	11Y	0	0	2	2	0	Not related
47	II	Female	12M	12Y	0	0	3	3	1	Not related
48	II	Female	12M	23Y	0	0	2	2	0	Not related
49	II	Female	12M	4Y	0	0	2	2	0	First cousins
50	II	Female	14M	3Y	0	0	3	3	1	First cousins
51	II	Female	14M	9Y	0	0	3	3	1	First cousin once removed
52	II	Female	14M	10Y	0	0	3	3	0	Not related
53	II	Female	14M	24Y	0	0	3	3	1	Not related
54	II	Female	15M	2Y	0	0	3	3	1	Not related
55	II	Female	15M	9Y	0	1	4	4	2	Second cousins
56	II	Female	15M	11Y	0	0	3	3	1	Not related
57	II	Female	15M	17Y	0	0	3	3	0	Not related
58	II	Female	15M	32Y	0	0	3	3	1	Not related
59	II	Female	17M	18Y	0	0	3	3	1	First cousins
60	II	Male	6M	4Y	0	0	3	3	0	Not related
61	II	Male	7M	9M	0	0	2	2	0	Not related
62	II	Male	7M	7Y	0	0	3	3	0	Not related
63	II	Male	7M	12Y	0	0	3	3	0	Not related
64	II	Male	7M	18Y	0	0	3	3	0	Not related
65	II	Male	8M	3Y	0	0	4	4	1	Not related
66	II	Male	8M	15Y	0	0	3	3	0	Not related
67	II	Male	9M	12M	0	0	3	3	1	Not related
68	II	Male	10M	9Y	0	1	3	3	1	Not related
69	II	Male	10M	13Y	0	0	3	3	1	Not related
70	II	Male	10M	14Y	0	0	3	3	1	Not related
71	II	Male	12M	12M	0	0	3	3	1	Not related
72	II	Male	12M	9Y	0	0	3	3	1	Not related
73	II	Male	12M	10Y	0	0	2	2	0	Not related
74	II	Male	12M	11Y	0	0	4	4	1	Not related
75	II	Male	13M	7Y	0	0	3	3	1	Not related
76	II	Male	14M	29Y	0	0	4	4	2	Not related
77	II	Male	15M	2Y	0	0	3	3	1	Not related
78	II	Male	15M	7Y	0	0	3	3	0	Not related
79	II	Male	16M	13Y	0	0	3	3	1	Not related
80	II	Male	17M	10Y	0	0	3	3	1	Not related
81	II	Male	18M	4Y	0	0	3	3	1	Not related
82	II	Male	22M	13Y	0	0	2	2	0	Not related
83	III	Female	20M	16Y	0	0	4	4	0	Not related
84	III	Female	2Y	2Y	0	0	3	3	0	Not related
85	III	Female	2Y	7Y	0	0	2	2	1	First cousins
86	III	Female	2Y	23Y	0	0	4	4	2	First cousins
87	III	Female	2Y	25Y	0	0	4	4	0	Not related
88	III	Female	2Y	27Y	0	0	2	2	0	Not related
89	III	Female	2Y	31Y	0	0	4	4	2	First cousins
90	III	Female	2Y	49Y	0	0	4	4	0	First cousin once removed
91	III	Female	3Y	4Y	0	0	4	4	0	Not related
92	III	Female	3Y	5Y	0	0	3	3	1	Not related
93	III	Female	3Y	9Y	0	0	4	4	1	Not related
94	III	Female	3Y	13Y	0	0	3	3	1	Not related
95	III	Female	3Y	15Y	0	0	3	3	0	Not related
96	III	Female	3Y	17Y	0	0	4	4	0	First cousins
97	III	Female	3Y	18Y	0	0	4	4	2	Not related
98	III	Female	3Y	19Y	0	0	3	3	1	Not related
99	III	Female	3Y	27Y	0	0	3	3	1	Not related
100	III	Female	3Y	31Y	0	0	4	4	2	First cousins
101	III	Female	4Y	4Y	0	0	3	3	1	Not related
102	III	Female	4Y	10Y	0	0	3	3	0	First cousins
103	III	Female	4Y	26Y	0	0	4	4	2	First cousins
104	III	Female	4Y	27Y	0	0	3	3	1	Not related
105	III	Female	4Y	32Y	0	0	3	3	1	Not related
106	III	Female	4Y	32Y	0	0	3	3	2	Not related
107	III	Female	5Y	6Y	0	0	3	3	1	Not related
108	III	Female	5Y	9Y	0	0	4	4	2	First cousins
109	III	Female	6Y	23Y	0	0	3	3	1	Not related
110	III	Female	7Y	13Y	0	0	3	3	1	Not related
111	III	Female	7Y	32Y	0	0	4	4	2	First cousin once removed
112	III	Female	7Y	38Y	0	0	3	3	1	Not related
113	III	Female	8Y	22Y	0	0	4	4	2	First cousins
114	III	Female	8Y	24Y	0	0	3	3	0	Not related
115	III	Female	8Y	39Y	0	0	2	2	0	Not related
116	III	Female	9Y	16Y	0	0	4	4	2	Not related
117	III	Female	9Y	21Y	0	0	4	4	2	Not related
118	III	Female	9Y	36Y	0	0	2	2	0	First cousins
119	III	Female	9Y	36Y	0	0	4	4	2	First cousins
120	III	Female	10Y	23Y	0	0	3	3	1	Not related
121	III	Female	11Y	28Y	0	0	3	3	1	Not related
122	III	Female	13Y	41Y	0	0	2	2	0	Not related
123	III	Female	14Y	34Y	0	0	3	3	1	Not related
124	III	Female	16Y	41Y	0	0	3	3	0	First cousin once removed
125	III	Female	17Y	34Y	0	0	2	2	0	Not related
126	III	Female	21Y	32Y	0	0	3	3	1	Not related
127	III	Male	18M	38Y	0	0	3	3	1	First cousin once removed
128	III	Male	18M	39Y	0	0	4	4	2	First cousins
129	III	Male	19M	7Y	0	0	2	2	0	Not related
130	III	Male	2Y	5Y	0	0	3	3	1	Not related
131	III	Male	2Y	12Y	0	0	4	4	2	First cousins
132	III	Male	2Y	16Y	0	0	3	3	0	Not related
133	III	Male	2Y	30Y	0	1	4	3	2	Not related
134	III	Male	2Y	30Y	0	0	4	4	2	First cousins
135	III	Male	2Y	33Y	0	1	4	3	2	Not related
136	III	Male	2Y	37Y	0	0	4	4	2	First cousins
137	III	Male	2Y	40Y	0	1	4	3	2	Not related
138	III	Male	2Y	43Y	0	1	4	3	2	Not related
139	III	Male	3Y	5Y	0	0	3	4	2	Not related
140	III	Male	3Y	7Y	0	0	2	2	0	Not related
141	III	Male	3Y	8Y	0	0	3	3	0	Not related
142	III	Male	3Y	10Y	0	0	3	3	1	Not related
143	III	Male	3Y	14Y	0	0	3	3	1	Not related
144	III	Male	3Y	14Y	0	0	3	3	1	Not related
145	III	Male	3Y	15Y	0	0	3	3	1	Not related
146	III	Male	3Y	17Y	0	0	4	4	1	First cousins
147	III	Male	3Y	24Y	0	0	4	4	2	First cousins
148	III	Male	3Y	26Y	0	0	4	4	2	First cousins
149	III	Male	3Y	32Y	0	1	3	2	2	Not related
150	III	Male	3Y	41Y	0	0	4	4	2	Not related
151	III	Male	3Y	43Y	0	0	4	4	2	First cousin once removed
152	III	Male	4Y	4Y	0	0	3	3	1	Not related
153	III	Male	4Y	10Y	0	0	4	4	2	Not related
154	III	Male	4Y	29Y	0	0	4	4	2	Not related
155	III	Male	5Y	7Y	0	0	4	4	2	First cousins
156	III	Male	5Y	15Y	0	0	2	2	0	First cousins
157	III	Male	6Y	20Y	0	0	3	3	2	Second cousins
158	III	Male	6Y	30Y	0	0	2	2	0	Not related
159	III	Male	7Y	29Y	0	0	3	3	0	Not related
160	III	Male	7Y	31Y	0	0	3	3	1	Not related
161	III	Male	7Y	31Y	0	0	4	4	2	First cousins
162	III	Male	11Y	12Y	0	0	3	3	2	Not related
163	III	Male	11Y	33Y	0	0	4	4	2	First cousins
164	III	Male	12Y	22Y	0	1	4	3	2	Not related
165	III	Male	12Y	35Y	0	0	4	4	2	First cousins
166	III	Male	12Y	40Y	0	0	3	3	0	Not related
167	III	Male	12Y	50Y	0	0	4	4	2	Not related
168	III	Male	13Y	16Y	0	0	3	3	2	First cousins
169	III	Male	13Y	23Y	0	0	4	4	2	First cousins
170	III	Male	13Y	26Y	0	0	4	4	0	Not related
171	III	Male	15y	27Y	0	0	4	4	2	First cousins
172	III	Male	15Y	30Y	0	0	4	4	0	First cousin once removed
173	III	Male	15Y	43Y	0	0	4	4	0	First cousin once removed
174	III	Male	17Y	32Y	0	0	4	4	2	First cousins
175	III	Male	18Y	33Y	0	0	4	4	2	Not related
176	III	Male	22Y	29Y	0	0	4	4	2	First cousin once removed
177	IV	Female	30Y	41Y	0	0	3	3	1	Not related
178	IV	Male	19Y	29Y	0	0	4	4	4	First cousins
179	IV	Male	20Y	33Y	0	0	4	4	2	First cousins
180	IV	Male	20Y	40Y	0	0	2	2	1	Not related
181	IV	Male	25Y	45Y	0	0	4	4	2	Second cousins
182	IV	Male	25Y	49Y	0	0	4	4	2	Not related
183	IV	Male	27Y	42Y	0	2	4	4	2	Not related
184	IV	Male	28Y	40Y	0	0	3	3	1	First cousins
185	IV	Male	28Y	40Y	0	0	4	4	2	First cousins
186	IV	Male	28Y	40Y	0	0	4	4	2	First cousins

Distribution of SMA patients in different groups of SMA is shown in Table 2. Type III SMA accounts for 50.53% of total cases.

**Table 2:** Results of genetic diagnosis of SMA patients.

Clinical type	Type I	Type II	Type III	Type IV	Total
Number	35	47	94	10	186
Proportion	18.81%	25.26%	50.53%	5.37%	100%

While exon 7 was absent in all SMA patients of all classes, exon 8 was present in 4 Type II, 6 Type III and 1 type IV SMA cases. In fact, in 94.08% (175/186) of the patients, homozygous deletion of both exons 7 and 8 of the *SMN1* gene was reported. Among these, 18.81% (35/186) of patients were diagnosed with SMA Type I, 25.26% (47/186) with Type II, 50.53% (94/186) with Type III, and 5.37% (10/186) with Type IV. In 5.9% (11/186) of the patients, homozygous deletion of the 7th exon and heterozygous deletion of 8th exon of the *SMN1* gene were detected (Table 3). There was no correlation between different SMA types and deletion types of exons 7 and 8 of *SMN1* gene (P value = 0.31).

**Table 3:** *SMN1* Exons copy numbers in patients with different clinical types of SMA.

Clinical types		Type I	Type II	Type III	Type IV	Total
Copy number						
EXON 7	0	35 (18.81%)	47 (25.26%)	94 (50.53%)	10 (5.37%)	186 (100%)
	1	0 (0%)	0 (0%)	0 (0%)	0 (0%)	0 (0%)
Total		35 (18.81%)	47 (25.26%)	94 (50.53%)	10 (5.37%)	186 (100%)
EXON 8	0	35 (18.81%)	43 (23.11%)	88 (47.31%)	9 (4.83%)	175 (94.08%)
	1	0 (0%)	4 (2.15%)	6 (3.22%)	0 (0%)	10 (5.37%)
	2	0 (0%)	0 (0%)	0 (0%)	1 (0.53%)	1 (0.53%)
Total		35 (18.81%)	47 (25.269%)	94 (50.53%)	10 (5.37%)	186 (100%)

**Figure 1:**
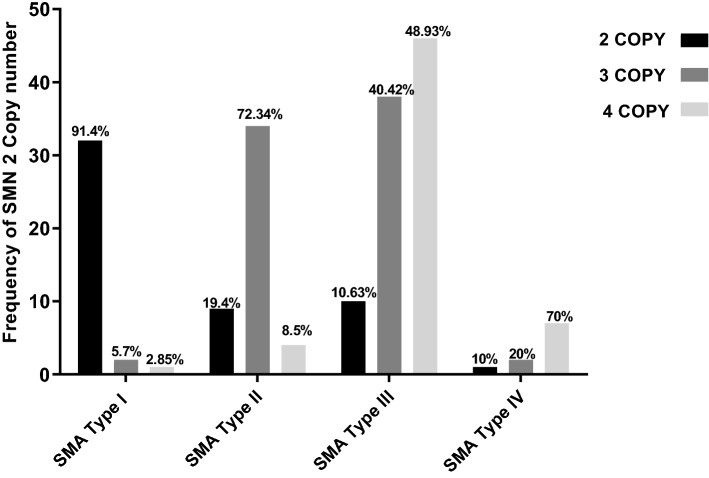
The percentage of individuals with various numbers of exon 7 of the *SMN2* gene.

Totally, 27.95% (52/186), 38.7% (72/186), and 28.49% (53/186) of patients had 2, 3, and 4 copies of exons 7 and 8 of the *SMN2* gene, respectively (Fig. 1). However, 9 patients showed different normal copy numbers of exons 7 and 8 of *SMN2* gene. Three out of nine patients showed 3 copies of exon 7 and 2 copies of exon 8, five out of nine patients showed 4 copies of exon 7 and 3 copies of exon 8 and one patient showed 3 copies of exon 7 and 4 copies of exon 8 of *SMN2* gene. In addition, 39.24% (73/186), 32.73% (59/186), 31.72% (53/186) and 0.53% (1/186) of patients had 0, 1, 2 and 4 copies of the exon 5 of the *NAIP* gene, respectively. The presence of two copies of *SMN2* gene was most common in type I patients, accounting for 91.42% (32/35) of these patients. The presence of 3 copies of *SMN2* was most common in type II patients, accounting for 72.34% (34/47) of patients. Finally, having 4 copies of this gene was most common in type III and type IV patients, accounting for 48.93% (46/94) and 70% (7/10) of patients, respectively.

Figure 1 shows the percentage of individuals with various number of *SMN2* gene copies.

**Figure 2:**
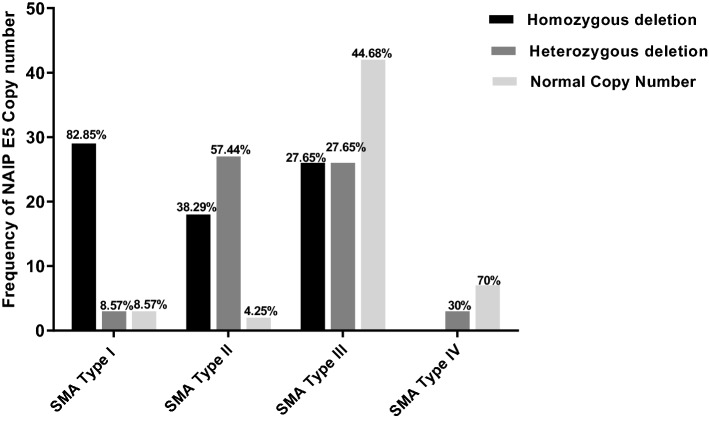
The percentage of individuals with various numbers of *NAIP* gene.

Figure 2 shows the percentage of individuals with various numbers of *NAIP* gene copies. There was a significant difference in the distribution of *NAIP* gene copy numbers among different types of SMA (× 2 = 69, P < 0.0001). All patients carrying deletion of two copies of *NAIP* gene had severe (type I) SMA, accounting for 82.85% (29/35) of patients. Having one copy of this gene was most common in type II patients, accounting for 57.44% (27/47) of patients. The presence of two copies of *NAIP* gene was most common in type III and type IV patients, accounting for 44.68% (42/94) and 60% (6/10), respectively (Fig. 2).

**Figure 3:**
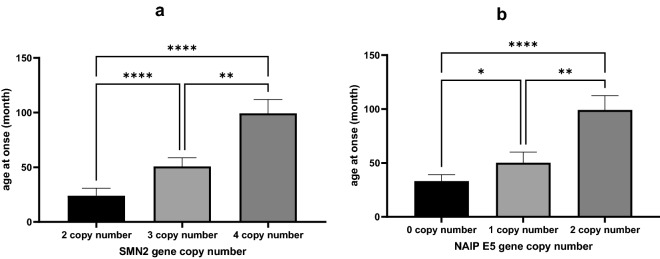
Relationship between copy numbers of exon 7 of *SMN2* (**a**) and *NAIP* (**b**) genes and age at onset of patients. A non-parametric Kruskal–Wallis test was used to identify significant association between the age at onset of patients and *SMN2* and *NAIP* genes copy number (* P value < 0.05, *** P value < 0.001 and **** P value < 0.0001).

The average age of onset for patients with 2 copies of *SMN2* gene (23.86 ± 50.14 month) was significantly lower than that of patients with 3 (50.75 ± 69 month) or 4 (99.25 ± 96.56 month) copies of *SMN2* (P < 0.0001) (Fig. 3a).

The average age of onset of SMA in patients with 0 copy of the *NAIP* gene (33.1 ± 51.98) was also less than that of patients with 1 (50.15 ± 75.81) and 2 (99 ± 96.65) copies (P < 0.0001) (Fig. 3b).

There was a significant difference in the distribution of *NAIP* gene copy numbers among different types of SMA. All patients carrying deletion of two copies of *SMN2* and *NAIP* genes had severe (type I) SMA.

Chi-square (2 × 3 contingency table) was performed to compare the distribution of *NAIP* E5 copy numbers between patient’s groups with/without parental relationship (not related vs. related groups). The analysis showed that there was significant difference (× 2 = 25.36, P < 0.0001) in the distribution of *NAIP* E5 copy numbers in patient’s groups regarding the parental relationship. In fact, 95 out of 115 (82.6%) of patients with no parental relationship had no or one *NAIP* E5 copy number and 20 (17.4%) of patients with no parental relationship had two *NAIP* E5 copy numbers. However, among the 71 patients with parental relationships, 27 patients (38%) had no *NAIP* E5 copy number, 34 patients (47.9%) had two *NAIP* E5 copy numbers and 10 patients (14.1%) had one *NAIP* E5 copy number.

There was also a strong significant correlation between copy numbers of *SMN2* and *NAIP* genes (R = 0.68, P < 0.0001) and the copy numbers of *SMN2* and *NAIP* genes had synergistic effect on SMA phenotype.

Integration of the data of the *SMN1*/*2* and *NAIP* genes showed 17 genotypes. Patients with genotype 0-0-3-3-1 (0 copies of *SMN1* (E7,8), 3 copies of *SMN2* (E7,8) and 1 copy of *NAIP* (E5)) were the most common genotype in this study (Table 4). Patients with 0-0-2-2-0 genotype were more likely to have type I SMA.

**Table 4:** Relationship between *SMN1* (E7and E8) -*SMN2* (E7and E8) -*NAIP* (E5) genotype and clinical phenotype of SMA.

Genotype	Case numbers (%)	Age of onset, month (mean ± SD)	Clinical classification (%)				Sex
			Type I	Type II	Type III	Type IV	
0-0-3-3-1	47 (25.26%)	54.48 ± 78.54	0 (0%)	22 (46.8%)	23 (48.9%)	2 (4.25%)	28 Female 19 Male
0-0-2-2-0	45 (24.19%)	21.42 ± 42.32	27 (60%)	9 (20%)	9 (20%)	0 (0%)	24 Female 21 Male
0-0-4-4-2	37 (19.89%)	113 ± 99	0 (0%)	1 (2.7%)	31 (83.78%)	5 (13.5%)	11 Female 26 Male
0-0-3-3-0	19 (10.21%)	41 ± 52.1	1 (5.26%)	9 (47.36%)	9 (47.36%)	0 (0%)	8 Female 11 Male
0-0-4-4-0	9 (4.83%)	74.75 ± 77.33	1(11.11%)	0 (0%)	8 (88.88)	0 (0%)	5 Female 4 Male
0-0-2-2-1	5 (2.68%)	55 ± 103.8	3 (60%)	0 (0%)	1(20%)	1(20%)	1 Female 4 Male
0-0-3-3-2	4 (2.15%)	82.5 ± 61.19	1 (25%)	0 (0%)	4 (75%)	0 (0%)	1 Female 4 Male
0-0-4-4-1	4 (2.15%)	23 ± 15.09	0 (0%)	2 (50%)	2 (50%)	0 (0%)	1 Female 3 Male
0-1-3-2-1	2 (1.07%)	10.5 ± 2.12	0 (0%)	2 (100%)	0 (0%)	0 (0%)	2 Female 0 Male
0-0-2-2-2	2 (1.07%)	1 ± 0	2 (100%)	0 (0%)	0 (0%)	0 (0%)	1 Female 1 Male
0-1-3-2-2	1 (0.53%)	36 ± 0	0 (0%)	1 (100%)	0 (0%)	0 (0%)	0 Female 1 Male
0-1-4-3-2	5 (2.68%)	48 ± 53	0 (0%)	0 (100%)	5 (100%)	0 (0%)	0 Female 5 Male
0-0-4-4-4	1(0.53%)	228 ± 0	0 (0%)	0 (0%)	0 (0%)	1 (100%)	0 Female 1 Male
0-1-4-4-2	1 (0.53%)	15 ± 0	0 (0%)	1 (0%)	0 (0%)	0 (0%)	1 Female 0 Male
0-2-4-4-2	1 (0.53%)	324 ± 0	0 (0%)	0 (0%)	0 (0%)	1 (0%)	0 Female 1 Male
0-1-3-3-1	1 (0.53%)	10 ± 0	0 (0%)	1 (0%)	0 (0%)	0 (0%)	0 Female 1 Male
0-0-3-4-2	1 (0.53%)	36 ± 0	0 (0%)	0 (0%)	1 (0%)	0 (0%)	0 Female 1 Male

**Figure 4:**
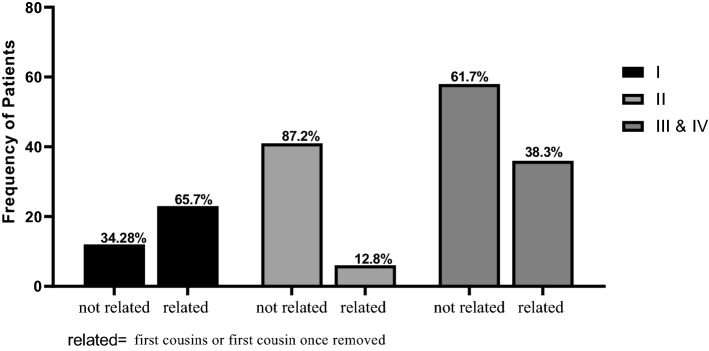
The distribution of clinical phenotypes (SMA subtypes) between patient’s groups with/without parental relationship. Chi-square (2 × 3 contingency table) was performed to compare the distribution of clinical phenotypes between patient’s groups (not related and related groups).

To compare the distribution of clinical phenotypes (SMA subtypes) between patient’s groups with/without parental relationship, patients’ group was divided into three subtypes I, II and III &IV. Chi square test in the 2 × 3 contingency table analysis provided evidence that there was significant difference (× 2 = 26.12, P < 0.0001) in the distribution of clinical phenotypes (SMA subtypes) in patient’s groups regarding the parental relationship. The frequency of type I patients was higher in patients with parental relationship (first cousins or first cousin once removed) while the frequency of patients with types II and III subtypes was higher in patients with non-consanguineous families (Fig. 4).

## Discussion

In the current study, we assessed *SMN1*, *SMN2* and *NAIP* copy numbers in a large population of Iranian patients with SMA. The majority of enrolled patients were born in non-consanguineous families which is consistent with high rate of normal carriers in Iranian population. A previous study in Iranian population estimated a carrier frequency of 5% in this population^11^. Consistent with this report, a more recent literature review has suggested higher frequency of heterozygous carriers of the *SMN1* mutations among Caucasian and Asian populations compared to the Black population^12^.

In our cohort of patients, all patients except for 11 cases had a homozygous deletion of exon 8 of *SMN1*. This finding is comparable with the findings in Chinese population^2^ and some other populations^13^.

The proportions of SMA cases with different numbers of normal *SMN2* copies were 2 copies in 53 (28.49%), 3 copies in 71 (38.17%) and 4 copies in 53 (28.49%). These values are significantly different from those reported by Fang et al. in Chinese population^2^. They reported the presence of 1–4 normal *SMN2* copies in 2 patients (4.8%), 14 (33.3%), 24 (57.1%) and 2 (4.8%) patients in their cohort, respectively^2^. Amara et al. have reported that 31.3% of Tunisian type I SMA patients carry one copy of *SMN2*, though all patients of other forms had a minimum of 2 copies^14^.

The proportions of SMA patients with different numbers of normal *NAIP* were 0 copy in 73 patients (39.24%), one copy in 59 patients (31.72%), 2 copies in 53 patients (28.49%) and 4 copies in one patient (0.5%). These figures are also different from Fang et al. report in Chinese population as authors reported 0–2 copies in 4 (9.5%), 26 (61.9%) and 12 patients (28.6%), respectively^2^. Moreover, *NAIP* has been reported to be absent in the majority of Tunisian SMA type 1 patients^14^.

Thus, there is significant difference in the copy number of mentioned genes among SMA patients of different populations. This difference might be due to the presence of some founder mutations in each population.

We also compared the copy numbers of *SMN2* and *NAIP* between four classes of SMA patients. These disease-modifying genes have been shown to influence age of onset of SMA patients. These two genes have been shown to be the most important modifier genes whose copy numbers can influence clinical course of SMA. Hassan et al. have shown that the combination of these genes has better performance in prediction of patients' prognosis than using CNVs of exon 7 of *SMN2* gene only. While CNVs of exon 7 of *SMN2* gene could predict response of patients to genetic therapy, deletion of exon 5 of *NAIP* gene alone could not predict severity of SMA^15^. Another study has shown that *NAIP* deletion is significantly related to the clinical severity of SMA and is a marker for prediction of SMA prognosis^16^. This finding has also been confirmed in our study, since all patients carrying deletion of two copies of *NAIP* gene had severe (type I) SMA.

Zhang et al.^17^ have determined five combined *SMN1*-*SMN2*-*NAIP* genotypes in their cohort of SMA patients with 0-3-1 genotype being the commonest one. Similarly, in our cohort of patients, 0-3-1 genotype had the highest frequency accounting for 26.19% of cases. Moreover, Zhang et al., have reported the synergistic effect of copy numbers of *SMN2* and *NAIP* genes on clinical course of SMA. They have demonstrated association between the combined *SMN1*-*SMN2*-*NAIP* genotypes with fewer copies and earlier disease onset and higher mortality in SMA patients^17^. Another study in Vietnamese population has shown association between copy numbers of *SMN2* and clinical severity of SMA. However, heterozygous *NAIP* deletion has been found commonly in SMA patients of this population in an independent manner from the clinical phenotype^18^. The latter finding is not consistent with our study, since we found association between copy numbers of both *SMN2* and *NAIP* genes and age of disease onset in Iranian population. Similar finding has been reported among Malaysian SMA patient^19^.

Taken together, the current study is the largest and the most comprehensive genetic analysis of Iranian patients that analyzed *SMN1*, *SMN2* and *NAIP* copy numbers simultaneously. This study also shows the spectrum of *SMN2* and *NAIP* copy numbers in Iranian SMA patients.

## Data Availability

The datasets generated and/or analysed during the current study are available in the Clinvar repository (https://www.ncbi.nlm.nih.gov/clinvar/?gr=0&term=smn).
